# Preclinical Detection of Variant CJD and BSE Prions in Blood

**DOI:** 10.1371/journal.ppat.1004202

**Published:** 2014-06-12

**Authors:** Caroline Lacroux, Emmanuel Comoy, Mohammed Moudjou, Armand Perret-Liaudet, Séverine Lugan, Claire Litaise, Hugh Simmons, Christelle Jas-Duval, Isabelle Lantier, Vincent Béringue, Martin Groschup, Guillaume Fichet, Pierrette Costes, Nathalie Streichenberger, Frederic Lantier, Jean Philippe Deslys, Didier Vilette, Olivier Andréoletti

**Affiliations:** 1 UMR INRA ENVT 1225, Interactions Hôtes Agents Pathogènes, Ecole Nationale Vétérinaire de Toulouse, Toulouse, France; 2 CEA, Institute of Emerging Diseases and Innovative Therapies (iMETI), Division of Prions and Related Diseases (SEPIA), Fontenay-aux-Roses, France; 3 UR892 Virologie et Immunologie Moléculaires Centre de Recherche de Jouy-en-Josas, Jouy-en-Josas, France; 4 Hospices Civils de Lyon –Laboratoire Diagnostic Maladies à Prions; CNRS, INSERM, UCB Lyon1, Centre de Recherche en Neurosciences de Lyon, BioRan, Bron, France; 5 VLA Weybridge, ASU, New Haw, Addlestone, Surrey, United Kingdom; 6 EFS-Nord de France, Quai de Jemmapes, Lille, France; 7 INRA, UMR 1282 Infectiologie et Santé Publique, Nouzilly, France; 8 Friedrich-Loeffler-Institut, Greifswald, Insel Riems, Germany; 9 Franklab, Montigny-le-Bretonneux, France; University of Alberta, Canada

## Abstract

The emergence of variant Creutzfeldt Jakob Disease (vCJD) is considered a likely consequence of human dietary exposure to Bovine Spongiform Encephalopathy (BSE) agent. More recently, secondary vCJD cases were identified in patients transfused with blood products prepared from apparently healthy donors who later went on to develop the disease. As there is no validated assay for detection of vCJD/BSE infected individuals the prevalence of the disease in the population remains uncertain. In that context, the risk of vCJD blood borne transmission is considered as a serious concern by health authorities. In this study, appropriate conditions and substrates for highly efficient and specific *in vitro* amplification of vCJD/BSE agent using Protein Misfolding Cyclic Amplification (PMCA) were first identified. This showed that whatever the origin (species) of the vCJD/BSE agent, the ovine Q_171_ PrP substrates provided the best amplification performances. These results indicate that the homology of PrP amino-acid sequence between the seed and the substrate is not the crucial determinant of the vCJD agent propagation *in vitro*. The ability of this method to detect endogenous vCJD/BSE agent in the blood was then defined. In both sheep and primate models of the disease, the assay enabled the identification of infected individuals in the early preclinical stage of the incubation period. Finally, sample panels that included buffy coat from vCJD affected patients and healthy controls were tested blind. The assay identified three out of the four tested vCJD affected patients and no false positive was observed in 141 healthy controls. The negative results observed in one of the tested vCJD cases concurs with results reported by others using a different vCJD agent blood detection assay and raises the question of the potential absence of prionemia in certain patients.

## Author Summary

Variant Creutzfeldt Jakob Disease (vCJD) cases were identified in patients who received blood products that had been prepared from donors who later developed the disease. The blood borne transmission of vCJD is a major concern for blood transfusion banks, plasma derived products manufacturers and public health authorities. A vCJD blood screening test would represent an ideal solution for identifying donors/blood donations that might be at risk. In this study, we describe a blood assay which is based on the *in vitro* amplification of vCJD agent by Protein Misfolding Cyclic Amplification (PMCA). In vCJD animal models (sheep and primate), the assay enabled the identification of infected individuals in a very early stage of the asymptomatic incubation phase. We also provide evidence of the high specificity and the high analytical sensitivity of this assay using blood samples from vCJD affected and healthy patients.

## Introduction

The emergence of variant Creutzfeldt Jakob Disease (vCJD) is considered a likely consequence of human dietary exposure to the Bovine Spongiform Encephalopathy (BSE) agent [1]. Both primate and sheep experimental models rapidly indicated that vCJD/BSE could be transmitted by blood transfusion [2], [3]. To date, three vCJD cases and one vCJD infected but asymptomatic individual have been identified in the United Kingdom (UK), in patients that received Red Blood Cell units from donors who developed symptoms of vCJD 17 months to 3,5 years after donation [4], [5]. More recently, one preclinical vCJD case was reported in the UK in a haemophiliac patient. This patient had been treated with one batch of FVIII that was manufactured using plasma from a donor who developed vCJD six months after donating blood [6].

The total number of vCJD clinical cases identified so far remains limited (225 patients worldwide at the time of writing). However the prevalence of vCJD infected and asymptomatic individuals in the BSE exposed population remains extremely uncertain [7]. A first retrospective analysis of stored lymphoid tissues indicated that vCJD prevalence in the UK could approach 1 out of 4000 individuals, though with wide confidence intervals [8]. More recently 32,441 appendix samples, collected during surgery on patients born between 1941 and 1985 were tested for abnormal prion protein accumulation. This study indicated a likely vCJD prevalence estimate of 1 in 2,000 in these age cohorts (95% Confidence Interval ranging from 1 in 3,500 to 1 in 1,250) [9].

In addition, human PrP transgenic mouse models indicated that the BSE agent can colonize lymphoid tissues without propagating to detectable levels in the brain and causing clinical disease. This suggests the possibility of silent carrying by vCJD infected individuals [10].

This data raised major concerns about the possible occurrence of inter-individual iatrogenic vCJD transmission in particular by blood and blood products. Despite a decade of efforts, there is currently no validated test that would allow the identification of vCJD infected but asymptomatic individuals or the screening of blood donations for the presence of the vCJD agent [11].

There is currently limited information related to the infectivity level and distribution in the blood components of vCJD affected patients. Bioassay testing of blood fractions from a single vCJD affected patient indicated an infectious titer of 4.45 ID per mL of blood which was approximately equivalent to the infectivity found in 1 µg of a reference vCJD brain sample [12]. Such low infectious titer makes the direct detection of prion in blood difficult to achieve. Like in various TSE animal models (mice, hamsters, sheep and cervids), a substantial part of the infectivity in this patient was associated with white blood cells (WBC) [13]–[16]. This suggests that WBC could be an appropriate target to detect endogenous vCJD agent presence in human blood.

Prions are primarily composed of multimers of a misfolded form (PrP^Sc^) of the host-encoded prion protein (PrP^C^). They propagate by recruiting and converting PrP^C^ into PrP^Sc^ and fragmentation of PrP^Sc^ multimers is thought to provide new PrP^Sc^ seeds for the conversion reaction. The Protein Misfolding Cyclic Amplification (PMCA) technology is aimed at replicating this phenomenon *in vitro*, allowing amplification of minute amounts of prions [17]. It facilitates the combining of a PrP^C^-containing substrate with previously undetectable amounts of PrP^Sc^ by repetitive cycles of incubation and sonication leading to amplification of abnormal PrP^Sc^. With this potential high sensitivity, PMCA has been proposed for prion detection in blood, and studies have been carried out in scrapie-infected hamsters and sheep that validated the concept that blood associated PrP^Sc^ can be amplified by PMCA [14], [18], [19]. However, despite the ability to amplify brain-derived vCJD agent by PMCA, the reported amplification performance was considered too limited for reliable detection in blood [20], [21].

In this study, we first identified PMCA substrate and conditions that allow highly efficient and specific amplification of the vCJD/BSE prions. We then show, using white blood cells as a template, that this method enables the identification of vCJD/BSE in asymptomatic experimental animals in the early phase of the incubation period.

## Methods

### Ethics statement

All animal experiments have been performed in compliance with institutional and French national guidelines, in accordance with the European Community Council Directive 86/609/EEC. Primates were housed and handled in accordance with the European Directive 2010/63 related to animal protection and welfare in research, under constant internal surveillance of veterinarians, in level-3 confined facilities entirely dedicated to prion research (agreement numbers A 92-032-02 for animal care facilities, 92-189 for animal experimentation), where cynomolgus macaque is the only housed animal species. Primates were placed in individual cages (a maximum of 20 cages per room) in six separate rooms, taking into account different parameters including the experiment they belong, their ages, their sex, their affinities to each other and their hierarchical status. Social enrichment was a constant priority, through individual activities and feeding according to the infectious risk. Animals were handled under anaesthesia (including blood sampling) to limit stress and avoid injury of handlers, and euthanasia (barbiturate overdose) was performed for ethical reasons when animals lost autonomy. The blood donor animals used in this study were included in experiments that were approved by the CETEA ethical committee (French Ethical Committee N°44, approvals 12-020).

Sheep were housed in level -3 containment animal care facilities (agreement numbers C-31-555-227 for animal care facilities, 31-09-555-47 for animal experimentation). The experimental protocol (oral challenge and blood collection) was approved by the Comité d'éthique Midi Pyrénées (ref MP/05/05/01/12).

Each healthy blood donor was individually informed and gave his/her written consent for using the collected samples in a scientific study. One vCJD blood sample included in the first panel of human blood samples was collected from a French patient at the clinical stage of the disease. According to French regulation, written informed consent for the use of this sample was obtained from the next of kin. Collection, storage and use of blood samples from vCJD patient included in this study was approved by national ethical authority (PHRC ref 2004-D50-353).

The use of the second human blood sample panel that was provided by the MRC Prion Unit, London, (United Kingdom) was approved by a UK national ethical committee (authorization number 03N/022). These samples were analyzed anonymously.

Finally, the experimental protocol on animals and the use of human samples was examined and approved by the INRA Toulouse/ENVT ethics committee.

### Sheep oral inoculation with BSE

TSE free sheep were produced in the Defra ‘New-Zealand flock’ which was a unique source able to provide animals that can be considered free from classical scrapie [22]. The animals included in our experiments were imported into France and housed in dedicated scrapie free facilities before their use in experiments. In all cases, PrP genotype was obtained by sequencing the Exon 3 of the *PRNP* gene as previously described [23], [24].

Four ARQ/ARQ sheep (6–10 months old) were orally challenged with 5 g equivalent of brain material (1% brain homogenate in glucose). The inoculum was prepared using brain from an ARQ/ARQ sheep experimentally challenged with cattle BSE. Animals were then observed until the occurrence of clinical signs and euthanized when exhibiting locomotor signs of the disease that impaired their ability to eat. White blood cells (WBC) from age and breed matched uninoculated TSE free ARQ/ARQ control animals (n = 60) were obtained by osmotic lysis of the buffy coat (one volume) with ACK solution (one volume) (NH_4_Cl 0,15 M, KHCO_3_ 1 mM, Na_2_EDTA 0,1 mM, pH 7.4) for 5 min RT. WBC were washed 3 times with 50 mL of PBS before being pelleted and stored at −80°C.

### Control and vCJD infected primates

Captive-bred 2.5 year-old male cynomolgus macaques (*Macaca fascicularis*) were provided by Noveprim (Mauritius). Primates were checked for the absence of common primate pathogens before importation, and handled in accordance with national guidelines. One animal (Macaque 6) was transfused with 40 mL of blood from a vCJD-infected primate sampled at the terminal stage of the disease. The other primates were intravenously inoculated with clarified supernatants (obtained by centrifugation at 1,500 g for 10 minutes after extensive sonication) derived from 10 or 100 mg of brains from BSE- or vCJD-infected primates. Such intravenous inoculation route is likely to mimic the contamination as it occurs in post-transfusion vCJD secondary cases.

Primate blood samples were drawn into sodium citrate and fractionated by centrifugation at 2,000 g for 13 minutes according to the techniques classically applied in human transfusion. WBCs were obtained by osmotic lysis of buffy coat (one volume) with Easy-lyse (Dako, 9 volumes) for 10 minutes RT. WBCs were washed three times with 50 mL of PBS. Animals were handled under anesthesia to limit stress, and euthanasia was performed for ethical reasons when animals lost autonomy.

The majority of the blood samples used for vCJD agent detection were obtained from archive collections. No influence was possible on the design of blood sampling plans. The possibility of collecting multiple samples from each animal was limited by ethical constraints (reduction of stress to the primates). All samples were encoded before dispatch and tested blind. None of the primates that were involved in this experiment suffered from the myelopathic syndrome recently described in primates challenged with human and primates blood products [25].

### Control and vCJD affected patients

In a first experiment related to human blood, WBC from 135 healthy volunteer human donors were prepared using the same protocol as in primates. In addition a vCJD blood sample collected in a French patient at clinical stage of the disease was tested. This blood sample was the same as the one used to measure vCJD infectivity in blood components by bioassay in a recently published study [12]. In this patient, vCJD was confirmed by both neuropathological examination and Western blot. All these samples were encoded before dispatch and testing.

In a second experiment, a panel of nine buffy coat samples was provided by the MRC Prion Unit (London, UK). This panel comprised material collected and prepared more than 10 years ago. It included three vCJD affected patients, and nine healthy patients. The blood volume that was used to prepare the buffy coat of each healthy patient varied between 3.5 to 8 mL. For one of the vCJD cases buffy coat samples were prepared using 3.5 mL of blood. In the two other vCJD cases the initial blood volume was undocumented. The nature of the anticoagulant used to collect the blood samples, the purity and the final number of WBC in the samples was not available. None of the vCJD samples that were included in this panel had been tested using the MRC vCJD blood assay described by Edgeworth et al. [26].

WBC were received as a frozen cell suspension (in 50 µL of PBS). They were re-suspended in 200 µL of PMCA amplification buffer before homogenization. The homogenates were then split in two and tested in parallel in INRA UMR 1225 (Toulouse, France) and INRA UR 892 (Jouy en Josas, France).

Brain material from vCJD (n = 4), sCJD (one MM1, one MM2, one VV1, one VV2, and one MV2) and Alzheimer's disease (n = 3) affected patients were obtained from the National Creutzfeldt-Jakob Disease Surveillance Unit (UK-Edinburgh) or from the French CJD national reference laboratories network [27].

For testing the inhibitory impact of red blood cells on vCJD amplification, red blood cells from a healthy human donor were separated from plasma and buffy coat by centrifugation (2000 g-13 min) and washed twice in PBS. Red cells were then submitted to two freezing/thawing cycles. The obtained red blood cell lysate was then used in the experiment.

### PMCA substrate

Transgenic mice lines that express PrP^C^ of different species were used to prepare substrates: tgBov (Bovine PrP, line tg110), tga20 (murine PrP), tg338 (ovine V_136_R_154_Q_171_ PrP), tgShXI (ovine A_136_R_154_Q_171_ PrP variant) and tg650 (Human Met129 variant of the human PrP). All but the bovine PrP expressing mice (tgBov) were established on the same mouse PrP^Ko^ background (Zurich I) [28]–[30]. In each of these mouse lines relative PrP expression level in the brain, by comparison to the natural host species, was described (tga20: 10-fold–tg338: 6–8 fold, tgBov/tg110: 8-fold, tgShpXI: 3–4-fold, tg650: 6-fold) [28], [31]–[34]

Mice were euthanized by CO_2_ inhalation and perfused (intra-cardiac) with PBS pH 7.4/EDTA 5 mMol (40–60 mL per mouse). The brains were then harvested and snap frozen in liquid nitrogen. 10% brain homogenate was prepared (disposable UltraTurax – 3 min) in 4°C PBS pH 7–7.65+0.1% Triton X100+ 150 mM NaCl (10% Weight/vol). The substrate was then aliquoted and stored at −80°C.

In order to check the PrP^C^ protein level in the PMCA substrates, total protein from an aliquot of each type of substrate was quantified by bicinchoninic acid (BCA, Pierce). Five µg of proteins were mixed with an equal volume of 2X Laemmli's buffer before Western Blotting and PrP^C^ immunodetection (see Western blot section below, supplementary figure 1).

**Figure 1:**
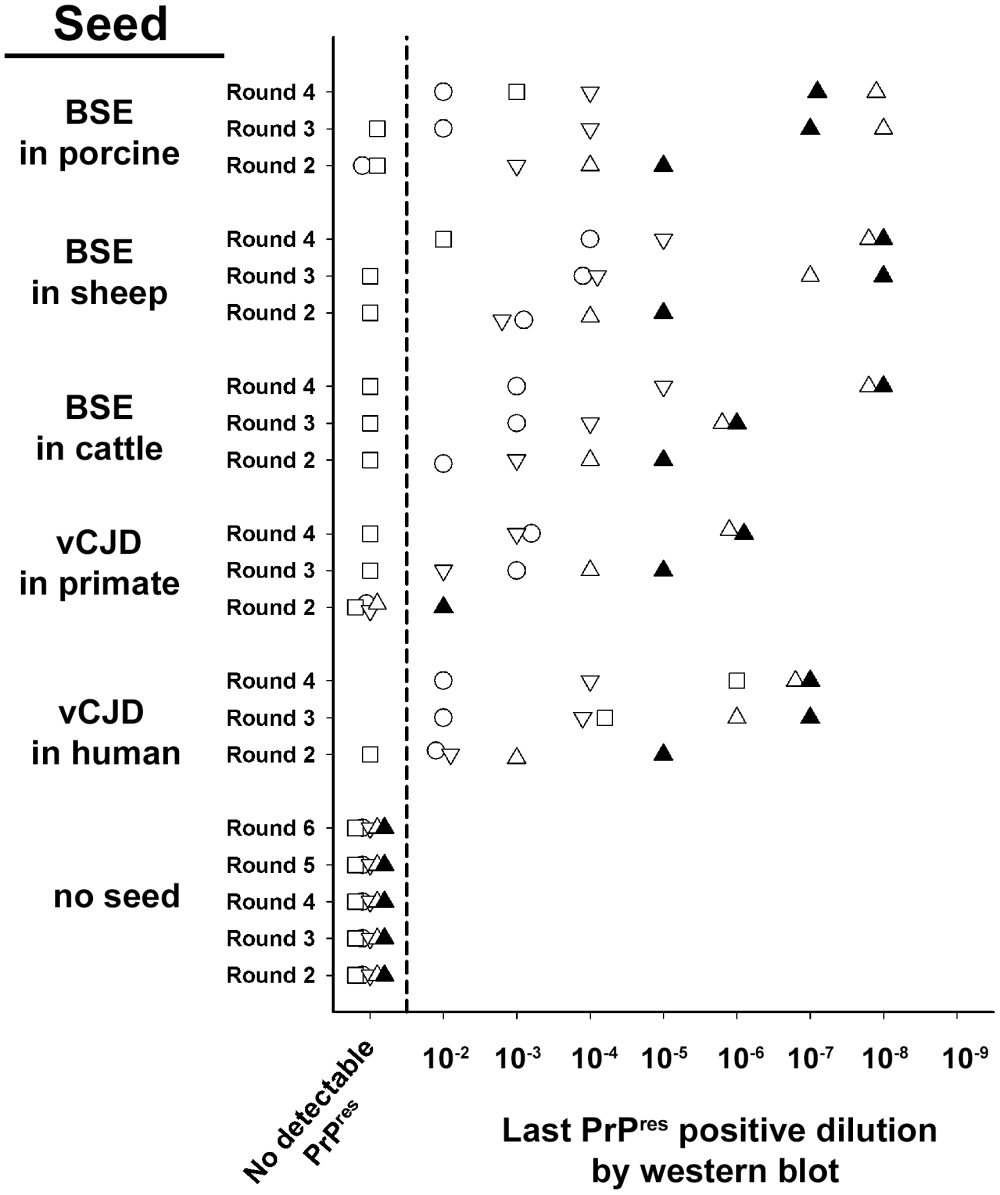
Relative performances of vCJD/BSE agent amplification by Protein Misfolding Cyclic Amplification using brain from transgenic mice expressing different species PrP sequences as substrate. PMCA reactions were seeded with ten-fold dilution series of vCJD/BSE brain material (10^−2^ to 10^−9^) from different species (human, *cynomologus* macaque, bovine, ARQ sheep and porcine). PMCA substrates were prepared using brain from transgenic mice over-expressing either human (methionine 129 variant, □), bovine (▿), murine (○) or sheep (VRQ variant:▵, ARQ variant:▴) Prion protein. Unseeded reactions were included as specificity control. PMCA reactions were then submitted to 2 to 6 amplification rounds each constituted with 96 cycles (30 s sonication-30 minutes incubation at 39.5°C) in a Misonix 4000 sonicator. After each round, (i) reaction products (1 volume) were mixed with fresh substrate (9 volumes) to seed the following round while (ii) a part of the same product was analysed by Western Blot (WB) for the presence of abnormal PK resistant PrP (PrP^res^ -antibody Sha31 epitope YEDRYYRE). For each round, the last dilution displaying a positive signal in WB is indicated on the graph.

### PMCA reaction and controls

The desired amount of WBC or Buffy coat were resuspended in 200 µL of 4°C PBS pH 7.4+150 mMoL NaCl+ 0.1 TRITON X100 and homogenized at high speed (Precess 48, Bertin, France). Samples were then spun down at 15000 g for 20 seconds and then stored at −80°C or used fresh. 7 µL of the seed were mixed with 63 µL of substrate in 0.2 mL ultrathin wall PCR tubes or 96 well microplates that contained five to eight 1 mm diameter silica/zirconium beads (Biospec Cat. No. 11079110z). Amplification was performed in a modified Misonix 4000 cup horn (see below), using a water recirculation system (39.5°C). The reaction tubes/microplates were then submitted to 96 cycles of 30 seconds sonication (power 70%) followed by a 29 minutes and 30 seconds incubation period.

After the PMCA round, 7 µL of the reaction product were added to a new tube containing fresh substrate and a new round (96 cycles) was performed. In order to limit the cross contamination risks that are linked to serial PMCA, procedures were employed that are similar to those in place for nested PCR. In particular, PMCA substrates, amplification and handling of amplified products were performed in different rooms using dedicated material.

On each PMCA run, a standard 1/10 dilution series (ovine BSE, 10% brain homogenate, 10^−5^ to 10^−9^ diluted) was included to check the amplification performance. A large batch of these controls was prepared and stored at −80°C as single use aliquots. Similarly unseeded controls (1 unseeded control for 5 seeded reactions) were included on each run.

A total of 68 PMCA runs were performed in the framework of this study. Contamination of some negative control reactions (false positives) was observed in 4 runs that had been performed in individual PCR tubes. In two of these runs, contamination was a likely consequence of a fault in the tube caps (obvious loss of reaction mixture in the tube). In two other cases the source of the contamination remained unclear, but the WB PrP^res^ pattern in false positive reactions was typical of a vCJD/BSE prion, making a cross contamination between tubes a likely explanation. No false positive reaction was observed in PMCA runs that were performed in 96 well PCR microplates. When a false positive was observed, the complete PMCA runs were discarded and restarted from the first amplification round.

### Misonix 4000× Sonicator modifications

Modifications consisted of the enlargement (5 mm inner diameter) of existing holes and creation of new holes for water recirculation in the crown surrounding the plate horn. These holes allowed a closed water circulation system in the horn delivering over 1.5 liters per minute of water. Permanent water re-circulation was ensured by a peristaltic pump (Watson Marlow 520 U) and deflectors were added to the horn to avoid water projection. The water circuit consisted of 10 metres of flexible tygon tube (diameter 9.2 mm) placed in a water bath. This system allowed the temperature of the water in the horn to return to its nominal value (39.5°C) within 20–40 seconds following the sonication burst and also maintained the water level in the horn at a constant level.

The bottom of the reaction tubes or 96 well microplates were positioned at a height of 2 mm above the horn plate and the water level in the horn was adjusted (before each PMCA round) to be at the same level as the reaction mixture in the tubes. Finally the acoustic protection box containing the sonicator horn was placed in an environment (temperature regulated room or incubator) maintaining the air temperature between 35°C and 40°C (limit of condensation).

### Western blot (WB) of abnormal PrP

PK resistant abnormal PrP extraction (PrP^res^) and Western blot were performed as previously described [35], using a commercial extraction kit (Biorad, France). For PMCA products the equivalent of 20 µL of reaction product were loaded on to each lane. PrP immunodetection was performed using either Sha31 monoclonal antibody (0,06 µg per mL, epitope: YEDRYYRE, amino acid 145–152) or 12B2 (4 µg/mL) (epitope WGQGG, amino acid sequences 89–93). Both Sha31 and 12B2 antibodies have been described in previous studies to bind the mouse, ovine, bovine, porcine and human PrP^C^ and PrP^res^ in WB [27], [36]–[39].

## Results

The first goal of the study was to identify a substrate and experimental conditions that together would enable a highly efficient PMCA amplification of vCJD/BSE agent. For that purpose, brain material from different transgenic mouse lines expressing ovine (A_136_R_154_Q_171_ and VRQ variants), bovine, human (Met_129_ variant) and murine PrP^C^ were used to prepare substrates. Reactions were then seeded with ten-fold dilution series of brain homogenate from vCJD/BSE-affected humans, primates, pigs, cows and sheep (figure 1).

**Figure 2:**
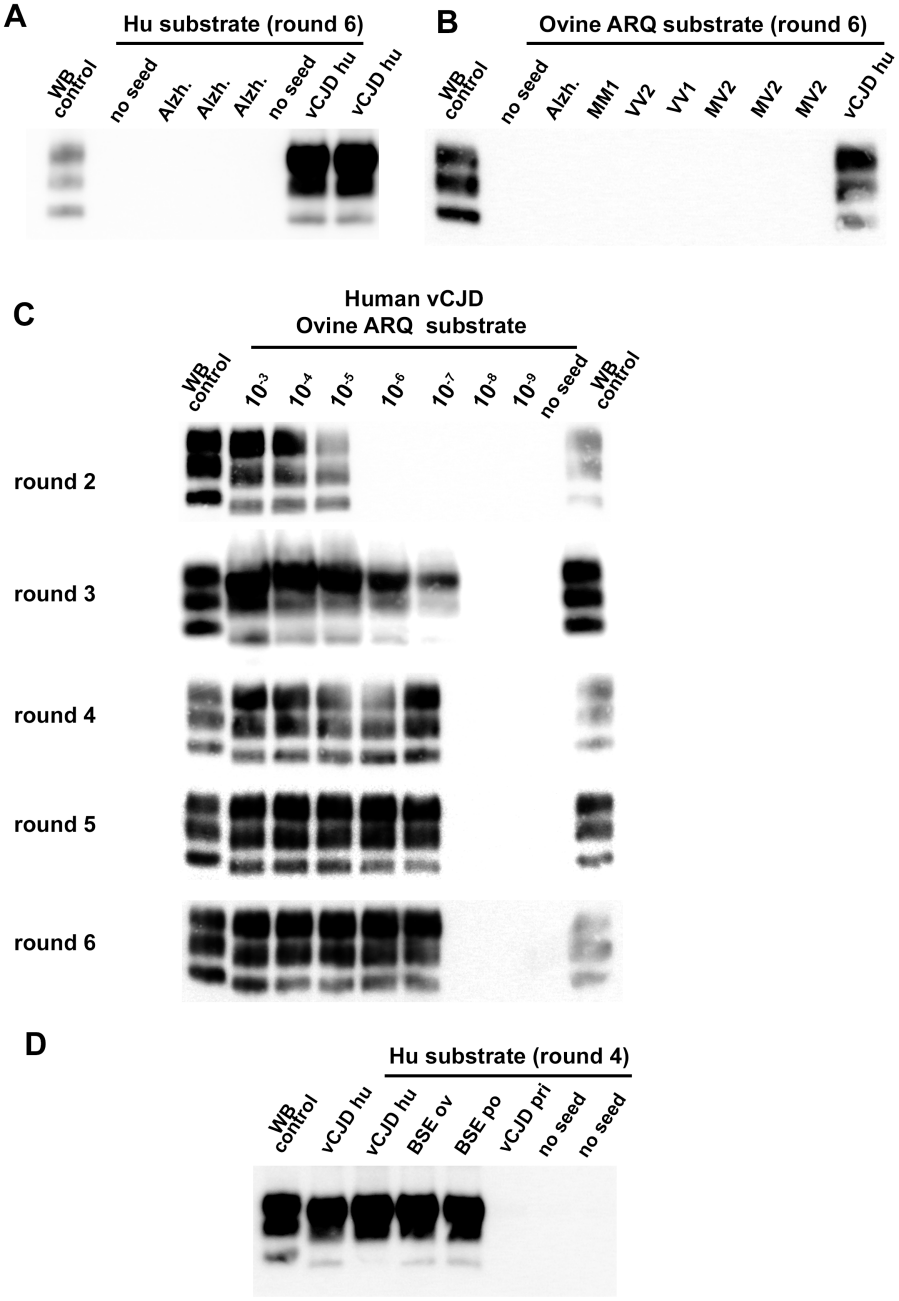
vCJD/BSE, sCJD and Alzheimer seeded Protein Misfolding Cyclic Amplification reactions using brain from transgenic mice as substrate. (**A–D**) WB PrP^res^ detection in PMCA reactions seeded with diluted 10% brain homogenates from human vCJD patient (vCJD Hu) or sporadic CJD (sCJD MM1, MV1, MV2, VV2 and VV1), ovine BSE (BSE Ov), porcine BSE (BSE po), or vCJD in primate (vCJD pri). A Scrapie isolate (WB control) and vCJD in human (vCJD Hu) isolate were used as positive control in an immunoblot (Sha31 anti PrP monoclonal antibody: epitope: YEDRYYRE, amino acid 145–152). Unseeded reactions (no seed) and reactions seeded with brain homogenate (10^−2^ diluted, 10% - frontal cortex) from 3 Alzheimer affected patients (Alzh.) were included as specificity controls. The nature of the PMCA substrate and the number of amplification rounds are indicated.

After six PMCA rounds, no PK resistant abnormal PrP (PrP^res^) could be detected by Western blot (WB) in un-seeded reactions (figure 1,2) or in those seeded with healthy brain material (data not shown). Whatever the substrate, no PrP^res^ was detected in reactions seeded with brain material from Alzheimer affected patients (figure 2A, B).

All the tested substrates allowed the amplification of vCJD/BSE, but displayed dramatically different detection limits. Whatever the origin (species) of the BSE/vCJD agent, the ovine PrP substrates (ARQ and VRQ) provided the best detection performances, *i.e.* positive for reactions seeded with a 10^−6^ to 10^−8^ dilution of the original brain homogenates (figures 1, 2C). No amplification was observed in ovine substrate reactions seeded with sCJD brain homogenates (figure 2B). All these results support the view that the homology of PrP amino-acid sequence between the seed and the substrate may not be the crucial determinant for vCJD/BSE agent PMCA amplification.

Strikingly, the capacity of the human PrP substrate to amplify the vCJD/BSE agent varied greatly according the infectious source species (figure 2D). Human vCJD, porcine BSE and ovine BSE prions were amplified using human PrP as a substrate but in contrast vCJD/BSE from cattle or primates was barely or not amplified.

**Figure 3:**
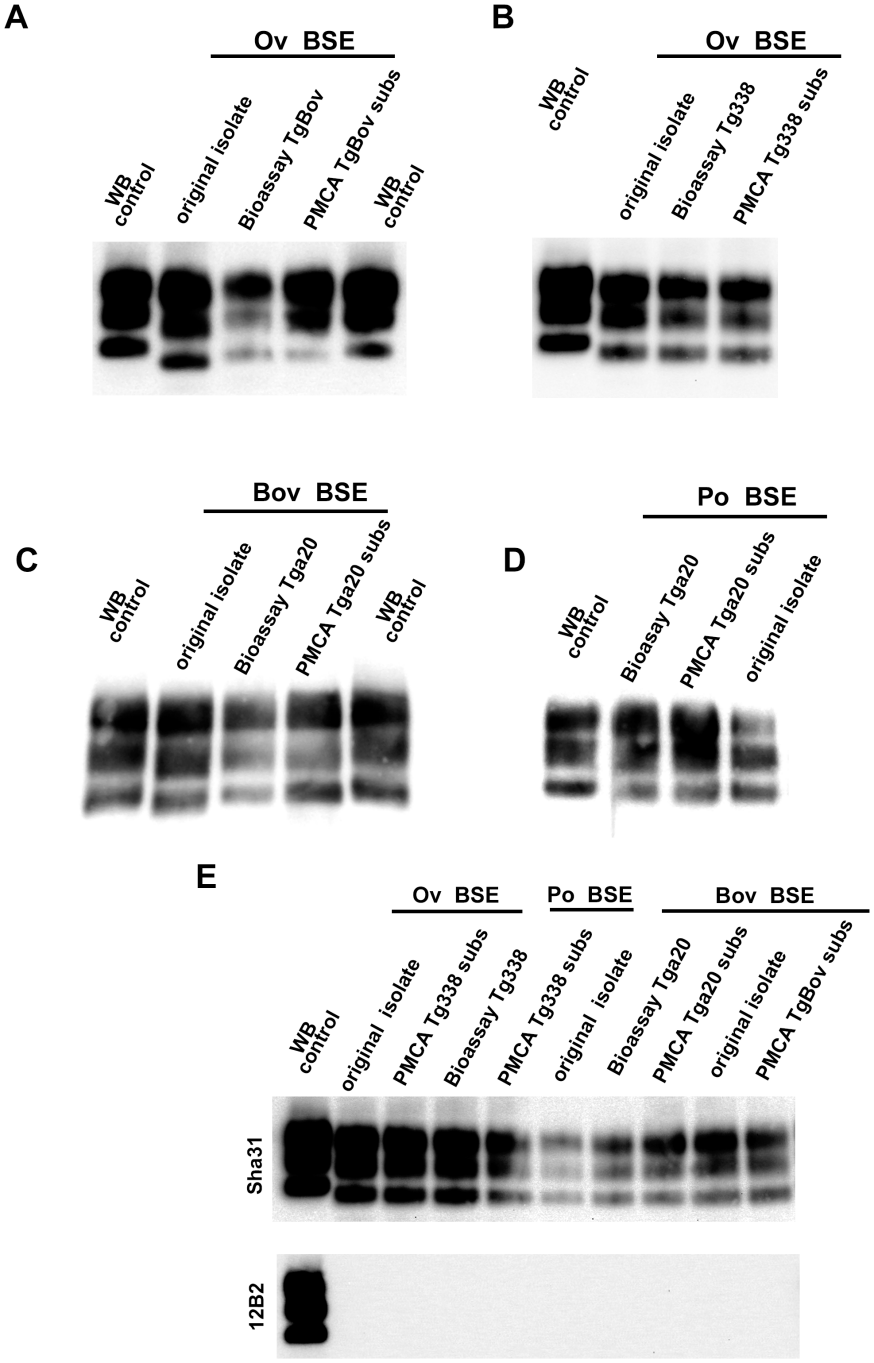
PrP^res^ western blot (WB) profile of BSE originating from various species before and after PMCA amplification. PMCA reactions were seeded with BSE brain material (10^−2^ dilution) from different species (bovine, sheep and porcine). PMCA substrates (sub) were prepared using brain from transgenic mice over-expressing the bovine (tgBov/tg110), the murine (tga20) or the sheep VRQ variant (tg338) Prion protein. PK digested abnormal PrP (PrP^res^) WB profile of (i) the original isolate used for seeding the PMCA reaction, (ii) the product of the PMCA reaction and (iii) the brain of a mouse intracerebrally inoculated with the seeding isolate and belonging to the line used to prepare the PMCA substrate, were compared. (**A–D**) WB PrP^res^ signature using Sha31 anti PrP monoclonal antibody (epitope: YEDRYYRE, amino acid 145–152). (**E**) Relative immunoreactivity of Sha31 (epitope: YEDRYYRE, amino acid 145–152) and 12B2 (epitope: WGQGG, amino acid sequences 89–93) anti PrP monoclonal antibodies. In vCJD/BSE, the 12B2's epitope is cleaved by abnormal PrP PK digestion process. For this experiment, duplicate of each samples were submitted to abnormal PrP extraction before migration on two different gels and Western blotted. One of the WB membranes was probed with Sha31 while 12B2 was used for the second. On each gel a scrapie in sheep isolate was used as control (WB control).

For all vCJD/BSE agent source/substrate combinations, the PrP^res^ WB pattern (glycoprofile and mobility) observed after PMCA amplification was indistinguishable from that observed in the brains of the transgenic mouse line used to prepare the PMCA substrate (figure 3A–D). In particular, the PrP^res^ obtained after PMCA displayed the same low/null immunoreactivity to 12B2 antibody (epitope WGQGG, amino acid sequences 89–93) as the original vCJD/BSE isolates (figure 3E). These results indicate that whatever the substrate, the amplified prion displays a PrP^res^ molecular signature consistent with BSE/vCJD.

**Figure 4:**
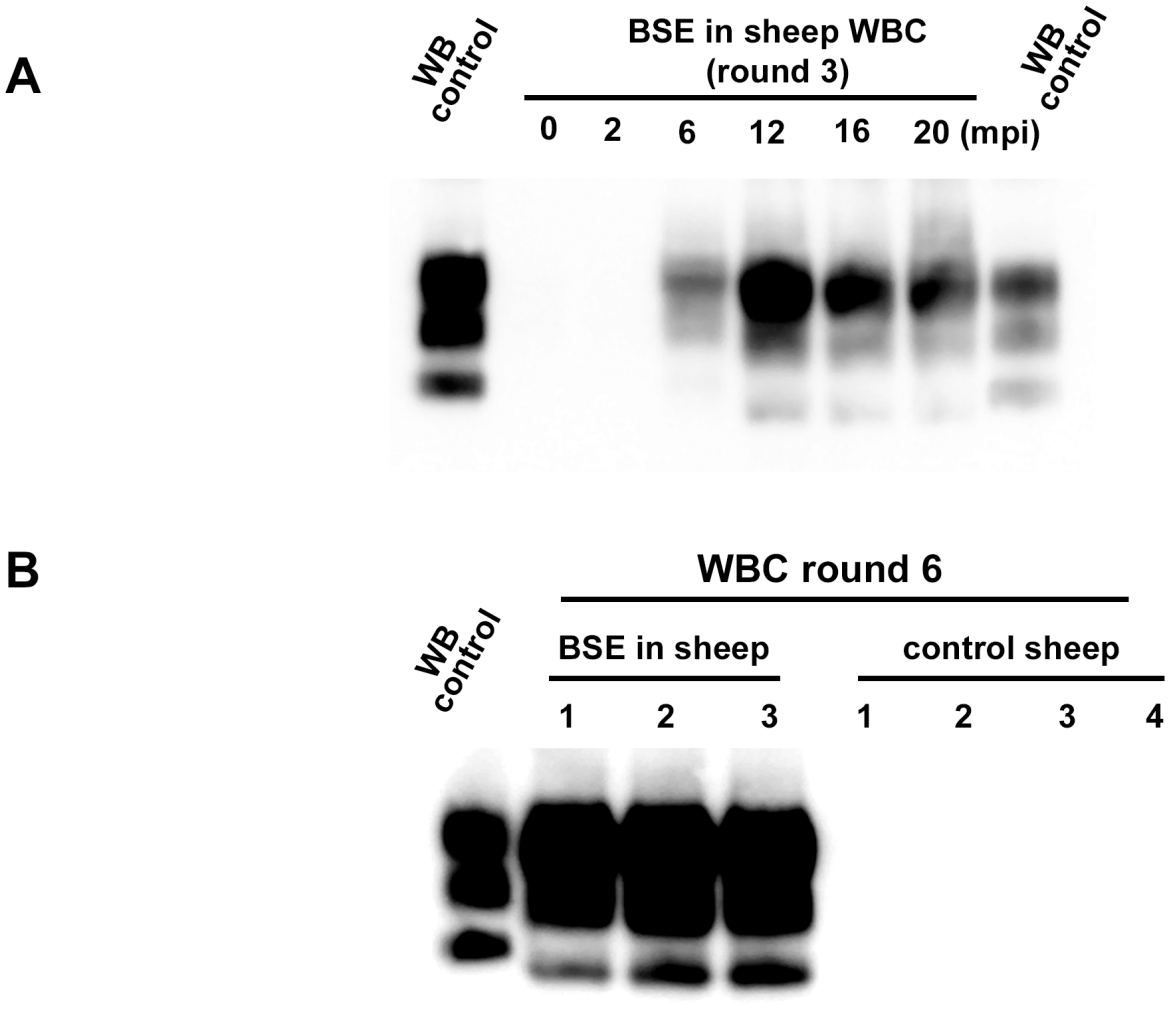
PrP^res^ detection in PMCA reactions seeded with white blood cells (WBC) from BSE infected and healthy sheep. WBC from BSE orally challenged and TSE free control ARQ/ARQ sheep were homogenised and used to seed PMCA reactions. Brain homogenate from ovine PrP transgenic mouse (ARQ variant) was used as PMCA substrate. Each sample was submitted to up to 6 rounds of amplification. Resulting PMCA products were analyzed by Western Blot (WB) for the presence of abnormal PK resistant PrP (PrP^res^ -antibody Sha31 epitope YEDRYYRE). On each gel a classical scrapie isolate (PK digested) was used as positive control (WB control). (**A**) In BSE orally challenged sheep (ARQ/ARQ), WBC prepared from blood collected at different time points (indicated as months post inoculation: mpi) of the incubation period were tested. The first clinical signs developed at 20 mpi. (**B**) BSE affected sheep (3 different individuals-20 mpi) and TSE free controls sheep (breed, genotype and age matched) were submitted to up to 6 PMCA rounds to check the specificity of the amplification.

In order to establish the capacity of the assay to detect endogenous vCJD/BSE agent in the blood, WBCs from 4 sheep orally challenged with BSE and 60 healthy control sheep were tested using the ovine ARQ substrate. In that experiment, the BSE infected sheep had developed disease 20 months post inoculation (mpi) (table 1). For all the symptomatic sheep, reactions seeded with WBC were shown to be positive after two PMCA rounds with a typical BSE PrP^res^ WB pattern. After four rounds, reactions seeded with WBCs collected at 6 mpi from some animals and at 12, 16 and 20 mpi from all animals were positive (table 1 and figure 4A). The WBC from the 60 TSE-free controls remained negative after 6 PMCA rounds (figure 4B).

**Table 1:** PrP^res^ detection in Protein Misfolding Cyclic Amplification (PMCA) reactions seeded with white blood cells from ARQ/ARQ sheep orally inoculated with BSE agent, collected at different time points of the incubation period.

	0 mpi				2 mpi				6 mpi				12 mpi				16 mpi				20 mpi			
	R2	R3	R4	R5	R2	R3	R4	R5	R2	R3	R4	R5	R2	R3	R4	R5	R2	R3	R4	R5	R2	R3	R4	R5
**Sheep 1**	-	-	-	-	-	-	-	-	-	-	-	-	-	2	4	4	3	4	4	4	4	4	4	4
**Sheep 2**	-	-	-	-	-	-	-	-	2	3	4	4	2	4	4	4	3	4	4	4	4	4	4	4
**Sheep 3**	-	-	-	-	-	-	-	-	-	2	4	4	1	4	4	4	4	4	4	4	4	4	4	4
**Sheep 4**	-	-	-	-	-	-	-	-	-	-	-	-	-	3	4	4	4	4	4	4	4	4	4	4

Four TSE free ARQ/ARQ sheep were orally challenged (before the age of 6 months) with 5 g of brain from BSE affected sheep. Blood was collected in these animals at 0, 2, 6, 12, 16 and 20 (clinical onset) months post inoculation (mpi). White blood cells (WBC) were obtained by osmotic lysis of the red blood cells. WBCs were then homogenized in PMCA buffer and homogenates were used to seed PMCA reactions in which the brain of transgenic mice that expressed the ARQ variant of the ovine PrP was used as substrate. Each sample was used to seed 4 independent reactions (two different runs onto 2 different sonicators). Five successive PMCA amplification rounds (R) were applied. After each amplification round, the number of PrP^res^ positive replicates (as assessed by Western blot) is indicated in the table. (-) indicated that all 4 replicates were negative.

(mpi): months post inoculation.

**Figure 5:**
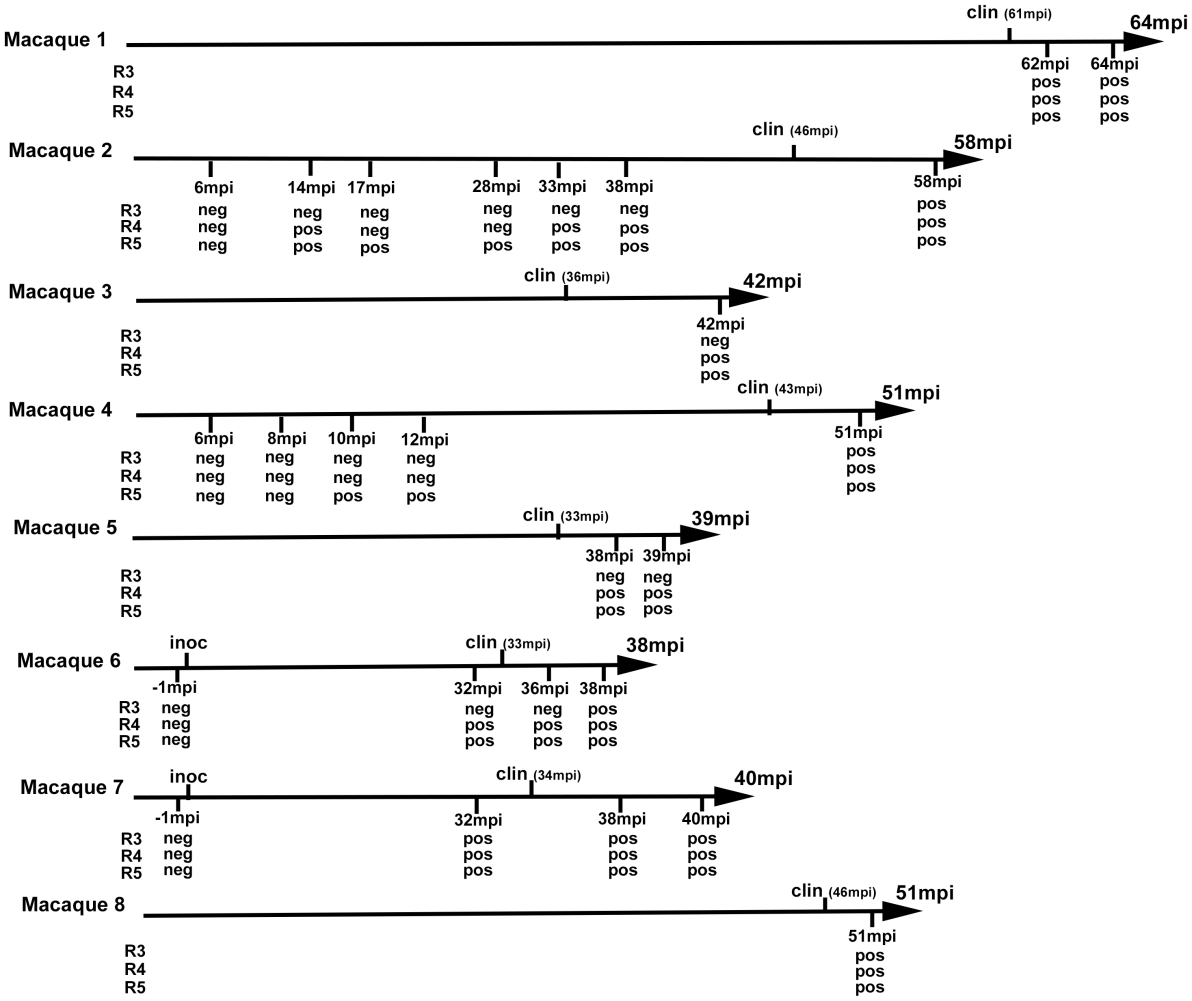
vCJD agent detection in the buffy coat of experimentally infected primates. Eight cynomologus macaques were intravenously challenged with vCJD brain homogenate or blood from a vCJD affected macaque (macaque 6). At different time points of the incubation period, blood was collected and buffy coat prepared. Clinical onset (clin) and time to euthanasia of the animals are indicated (upper label on arrows) as months post inoculation (mpi). The buffy coat samples were used (as homogenates 1/100 diluted in PMCA buffer) to seed PMCA reactions in which brain homogenate from ovine PrP transgenic mouse (ARQ variant) was used as substrate. Each sample was submitted to 6 rounds of amplification each comprising 96 cycles (30 s sonication-30 minutes incubation at 39.5°C) in a Misonix 4000 sonicator. PMCA products were analyzed by Western Blot (WB) for the presence of abnormal PK resistant PrP (PrP^res^ -antibody Sha31 epitope YEDRYYRE). Samples were received encoded and tested blind. The time point corresponding to blood samples (months post inoculation) that were tested and the results of PrP^res^ WB detection in PMCA reactions are indicated (under arrow). No positive WB result was observed before the third PMCA round. No additional positive result was observed after 5 PMCA rounds.

These promising results enabled us to test blood samples collected from vCJD-infected primate experiments (figure 5). This model is considered to be the closest to infection in humans [2]. Buffy coat (BC-n = 33) and WBC (n = 14) obtained by red cell lysis of BC from vCJD-infected (n = 8) and control (n = 15) *cynomolgus* macaques were tested. The animals had been challenged by the intravenous route using either brain homogenate (n = 7) or blood from a vCJD-affected primate and developed the disease with incubation periods ranging from 33 to 61 months (figure 5). All samples were encoded before dispatch and were tested blind.

**Figure 6:**
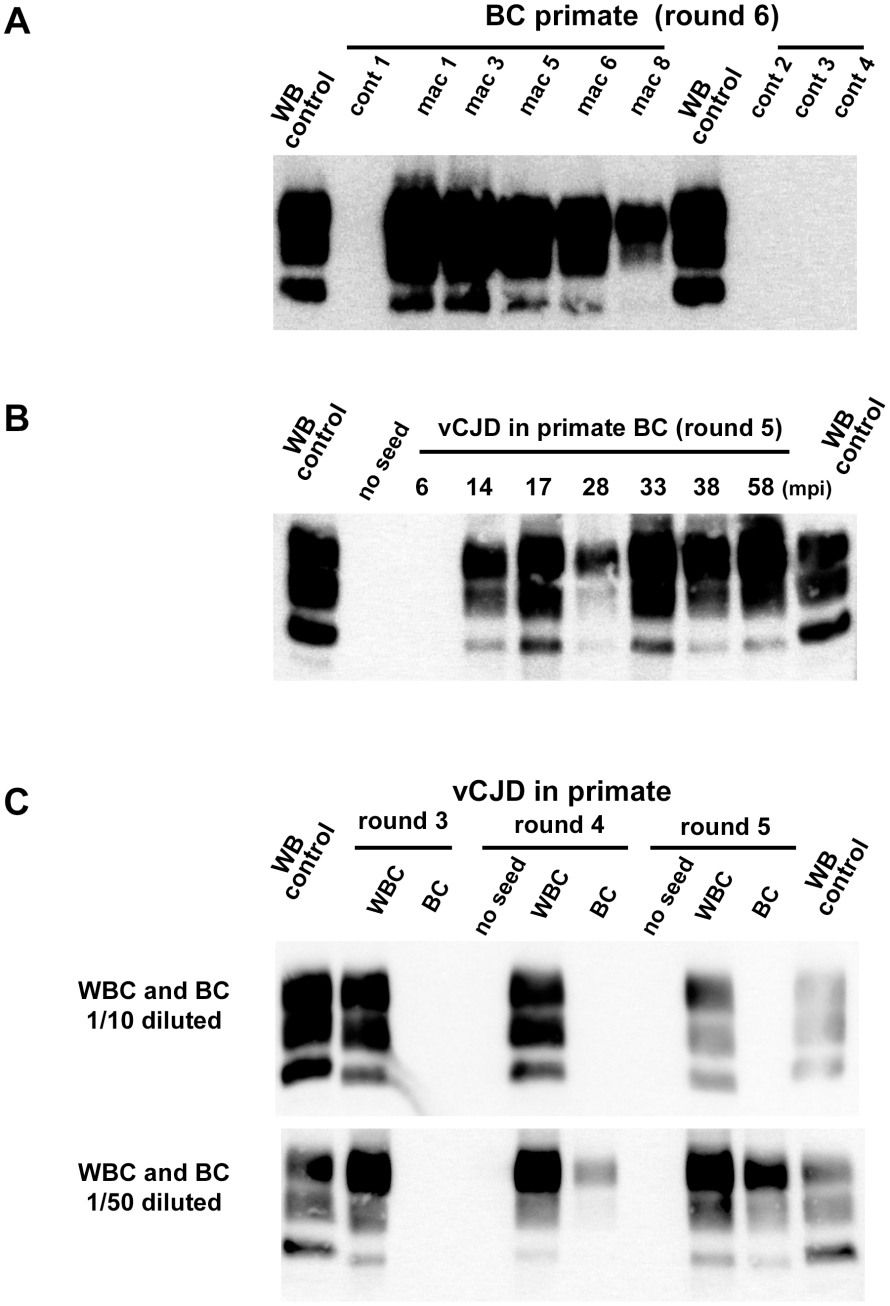
PrP^res^ in PMCA reactions seeded with blood samples from vCJD infected and healthy primates. Buffy coat samples (BC) or white blood cells samples (WBC) were prepared using blood volume collected from vCJD infected primates. Homogenized BC and WBC were used to seed PMCA reactions. In all PMCA brain homogenate from ovine PrP transgenic mouse (ARQ variant) was used as substrate. Each sample was submitted to 6 rounds of amplification each comprising 96 cycles (30 s sonication-30 minutes incubation at 39.5°C) in a Misonix 4000 sonicator. The resulting PMCA products were analyzed by Western blot (WB) for the presence of abnormal PK resistant PrP (PrP^res^ -antibody Sha31 epitope YEDRYYRE). On each gel a classical scrapie isolate (PK digested) was used as a positive control (WB control). (**A**) BC from four different unchallenged cynomolgus macaques (cont) and 5 different vCJD affected primates (see figure 5) were blindly tested. The WB corresponds to the original one performed before decoding the samples after 6 PMCA rounds (3 controls and 4 infected animals). (**B**) BC samples (collected between 2005 and 2012) were prepared at different time points of the incubation period (indicated as mpi) in a vCJD inoculated primate (intravenous route, macaque 2 in figure 5). The animal developed clinical signs at 46 mpi and was euthanized at 58 mpi. (**C**) WBC and BC were prepared from the same blood sample collected in a clinically affected primate (macaque 6–38 mpi). Diluted WBC and BC were used to seed PMCA reactions (see table 2).

After 4 PMCA rounds, blood from all the clinically affected primates was positive (figures 5, 6A). All the reactions seeded with BC or WBC (n = 17) from unchallenged primates remained negative after 6 PMCA rounds (figure 6A).

In four vCJD-infected primates (macaques 2, 4, 6 and 7), BC had been collected at different times during the asymptomatic phase of the incubation period. The reactions seeded with BC collected from 10 mpi to 14 mpi (more than 32 months before clinical onset) were positive after five PMCA rounds (figures 5, 6B). These data indicate that vCJD infection can be detected in the early preclinical stage in primates.

**Figure 7:**
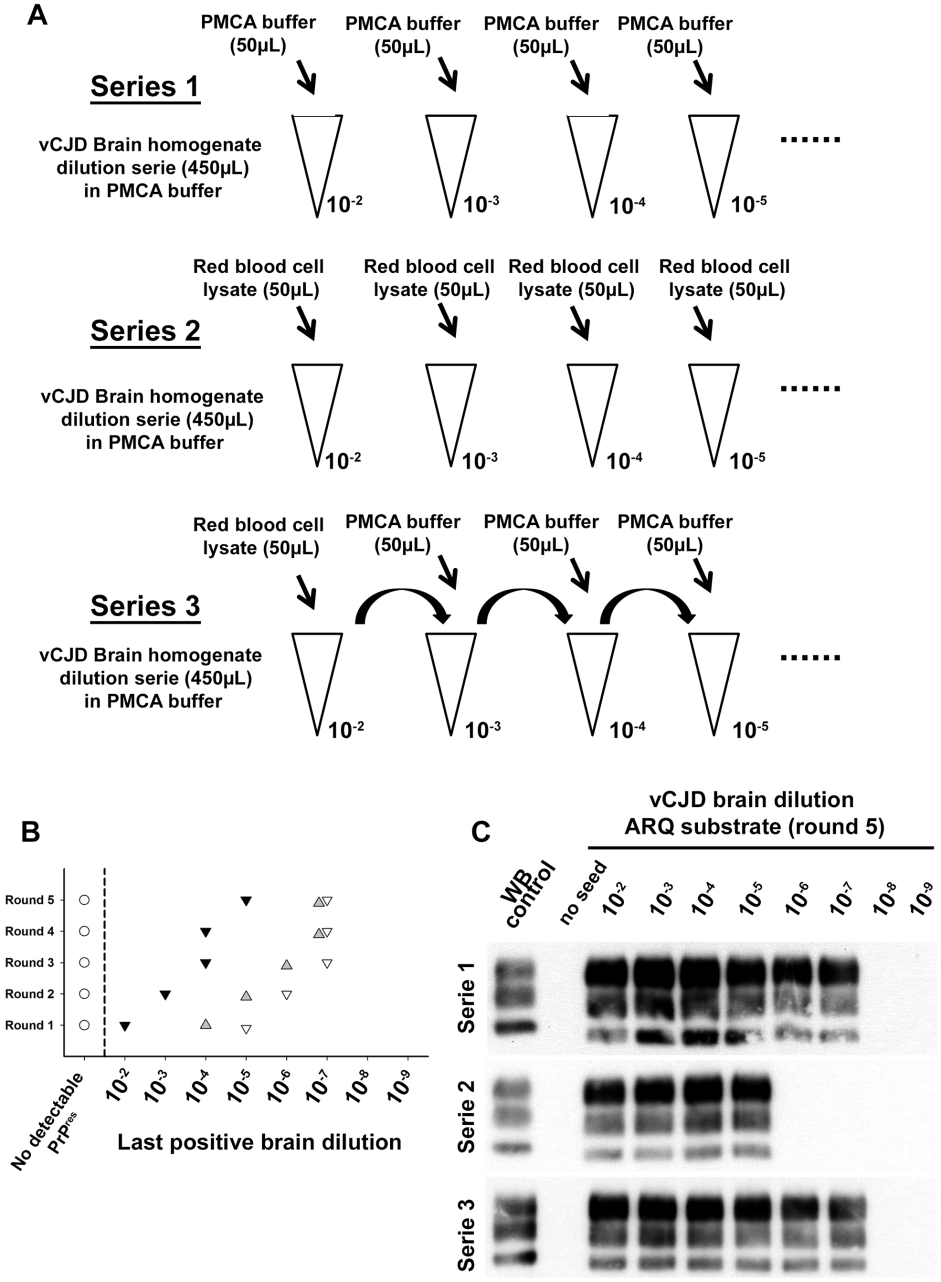
Red blood cell and inhibition of PMCA vCJD amplification. (A) Two vCJD brain homogenate (human) dilution series (1/10 dilution, 10^−2^ to 10^−9^) were prepared (aliquots of 450 µL). Each aliquot also contained 50 µL of PMCA buffer (series 1: ▿) or Red blood cell lysate (human) (series 2:▾). A third series (series 3: 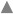) was prepared starting from a 10^−2^ dilution of the vCJD brain homogenate (900 µL) in which 100 µL of red blood cell lysate have been added. In each aliquot (450 µL) of dilution series 3, 50 µL of PMCA buffer were added. The three dilution series were then used to seed PMCA reactions (7 µL of seed) in which ovine PrP expressing mice (ARQ variant) was used as substrate (63 µL). Five successive rounds of PMCA were performed. After each round PrP^res^ detection was carried out in PMCA reactions by Western blot (Sha31 anti PrP monoclonal antibody: epitope: YEDRYYRE, amino acid 145–152). Ten unseeded controls (○) were included in the experiment. The results of the PMCA amplification after each round are presented in graph (**B**). WB corresponding to the fifth round of amplification is presented as an illustration (**C**).

The comparison of PMCA reactions seeded with BC and WBC prepared from the same blood samples suggested the presence of amplification inhibitor(s) in the BC (figure 6C and table 2). The negative effect of red blood cell presence on the vCJD amplification by PMCA was demonstrated by spiking a vCJD brain dilution series with red blood cell lysate (figure 7A). The addition of red blood cells resulted in a lack of amplification in reactions seeded with low dilutions of vCJD brain material (figure 7B, C). This loss of sensitivity in the vCJD amplification was not compensated by a higher number of PMCA rounds. However this inhibitory effect was compensated for/attenuated by diluting the red blood cell tainted seed in PMCA buffer prior to amplification. To limit inhibition, BC had to be diluted at least fifty-fold before being processed (figure 6C, table 2). This phenomenon could impact on the final sensitivity of the assay when applied to BC samples and could explain some of the negative results obtained in samples from asymptomatic but infected primates (figure 5 -macaque 2).

**Table 2:** PrP^res^ detection results in PMCA reactions seeded with white blood cells (WBC) or buffy coat (BC) from *Cynomologus* macaques clinically affected with vCJD.

		1/10				1/50				1/100			
		R2	R3	R4	R5	R2	R3	R4	R5	R2	R3	R4	R5
**Macaque 6**	**WBC**	−	+	+	+	−	+	+	+	−	+	+	+
	**BC**	−	−	−	−	−	−	+	+	−	−	+	+
**Macaque 8**	**WBC**	−	+	+	+	−	+	+	+	−	−	+	+
	**BC**	−	−	−	+	−	−	+	+	−	−	+	+

*Cynomologus* macaques were intravenously challenged with (i) blood from a vCJD affected macaque (macaque 6) or (ii) human vCJD brain homogenate (macaque 8). WBC and BC were prepared from the same blood sample (5 mL citrate dextrose anticoagulant) collected from the clinically affected primate (just prior euthanasia); macaques 6 and 8 were respectively euthanased at 38 months post inoculation (mpi) and 51 mpi and sampled at that time (see figure 4).

WBC and BC were homogenized and then diluted 1/10, 1/50 and 1/100 in PMCA buffer before seeding PMCA reactions. Brain from transgenic mice that expressed the ARQ variant of the ovine PrP was used as substrate. Samples were submitted to 5 successive rounds (R) of amplification. PrP^res^ detection was carried in each PMCA reaction by Western blot (WB) using Sha31 anti PrP antibody (epitope epitope: YEDRYYRE, amino acid 145–152).

**Figure 8:**
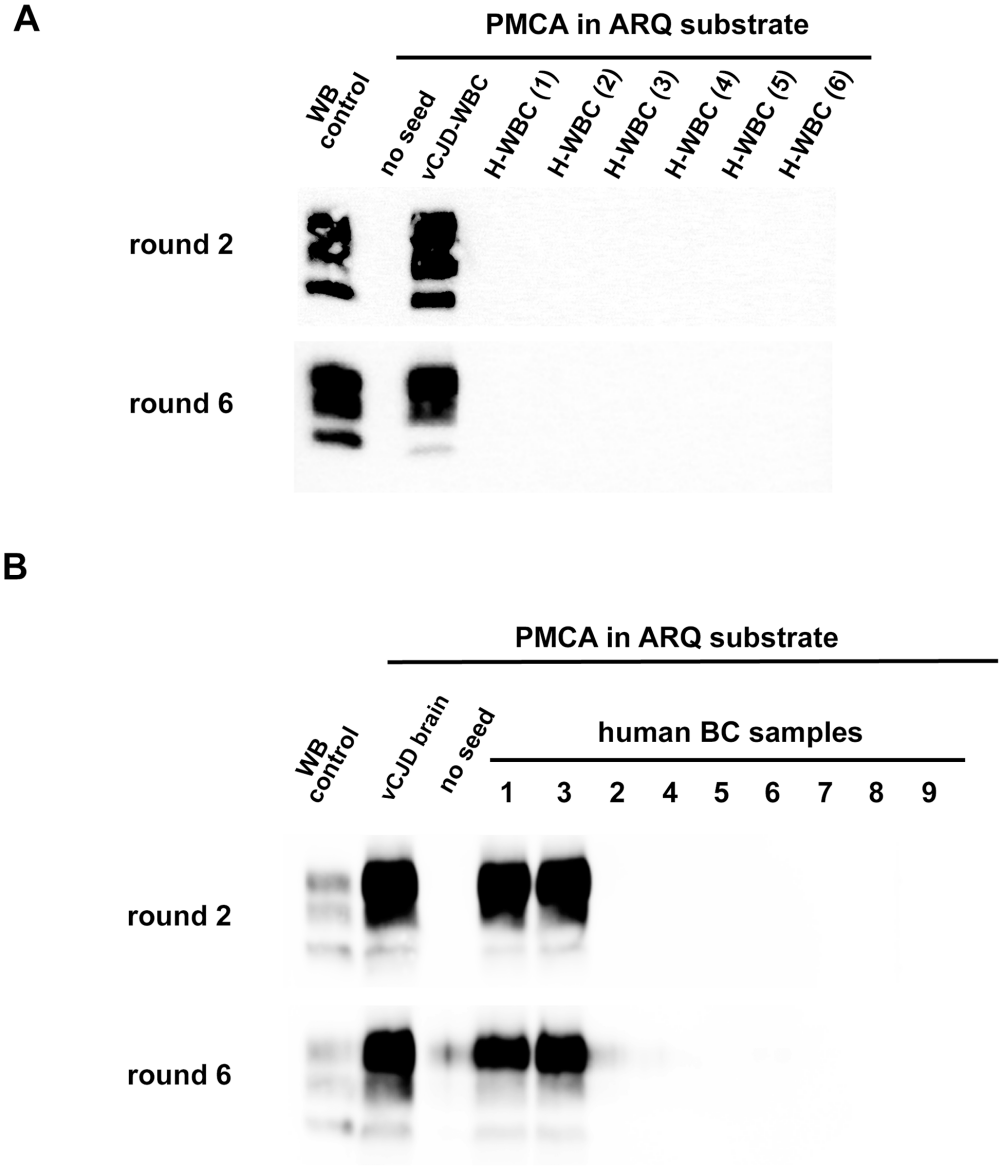
PrP^res^ in PMCA reactions seeded with WBC or BC from a vCJD affected patients and healthy controls. (**A**) WBCs from a French vCJD affected patient (vCJD-WBC) and healthy controls (H-WBC) were used to seed serial PMCA amplification (six rounds). PMCA controls included unseeded reactions (no seed). (**B**) Similarly, nine human buffy coat samples (received from the MRC Prion Unit (London, UK) were used to seed serial PMCA amplification (six rounds). The panel included three vCJD affected patients (sample 1, 3 and 8) and six healthy controls. A vCJD brain homogenate (10%, 10^−8^ diluted) was used as positive amplification control. In all cases brain homogenate from ovine PrP transgenic mice (ARQ variant) was used as substrate. PMCA products were analyzed by Western blot (WB) for the presence of abnormal PK resistant PrP (PrP^res^ -antibody Sha31 epitope YEDRYYRE). On each gel a classical scrapie isolate (PK digested) was used as positive control (WB control).

The results obtained in vCJD infected primates allowed access to a first panel of human blood samples that included WBCs from one French vCJD affected patient and 135 healthy controls. Samples were received encoded and tested blind. After 6 PMCA rounds, no PrP^res^ was detected in reactions seeded with WBC from human healthy controls (figure 8A). In contrast, two PMCA rounds (figure 8A) were sufficient to detect PrP^res^ in reactions seeded with the vCJD affected patient's WBC.

In order to test additional samples from vCJD infected patients we next contacted the MRC Prion Unit (London UK). They provided us with a panel of nine buffy coat samples that included three vCJD cases (confirmed by neuropathology and Western blot) and six healthy controls. The samples were received encoded and tested blind in two laboratories (UMR INRA ENVT 1225, Toulouse and UR 982 Jouy en Josas) using the same methodology.

**Figure 9:**
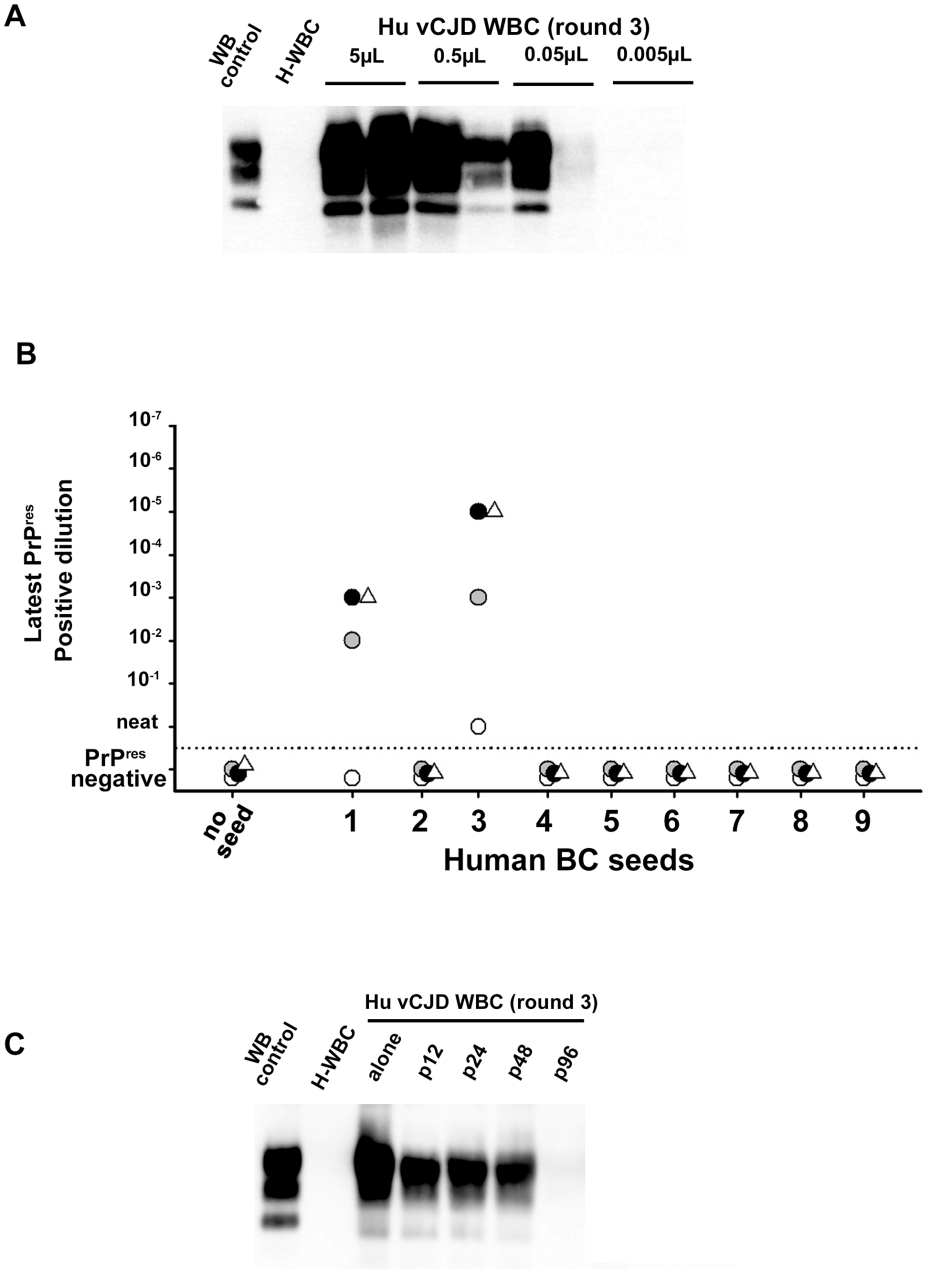
Analytical sensitivity of endogenous vCJD agent PMCA detection in the blood of affected patients. 1/10 dilution series (in PMCA buffer) were prepared using (**A**) WBC homogenate from one French vCJD affected patient and (**B**) buffy coat homogenates from two UK vCJD affected patients. These dilutions series were used to seed serial PMCA amplifications (up to 6 rounds) using brain homogenate from ovine PrP transgenic mouse (ARQ variant) as substrate. After each round, PMCA products were analyzed by Western Blot (WB) for the presence of abnormal PK resistant PrP (PrP^res^ - antibody Sha31 epitope YEDRYYRE). On each gel a classical scrapie isolate (PK digested) was used as positive control (WB control). (**A**) WBC homogenate from the French vCJD affected patient (Hu vCJD WBC) and healthy patients (H-WBC) were submitted to three amplification rounds. Each dilution was tested in duplicate. The equivalent whole blood amount used to seed the reactions is indicated in the figure. (**B**) PrP^res^ WB detection after the first (white circle), the second (grey circle), the third (black circle) and the sixth (white triangle) round of PMCA. Reactions were unseeded (no seed) or seeded with serial 1/10 dilution of the nine BC samples provided by the MRC unit (London, UK). (**C**) WBC from the French vCJD affected patient was tested either alone or after pooling with WBC from 11 (p12), 23 (p24), 47 (p48) or 95 (p96) healthy controls. The WBC homogenates used to prepare pools were equivalent to 50 µL of starting whole blood. Reactions seeded with WBC from healthy controls (H-WBC) were included as controls.

In both laboratories, the PMCA results were identical. After six PMCA rounds no PrP^res^ was detected in reactions seeded with BC from the healthy controls. Two PMCA rounds were sufficient to detect PrP^res^ in PMCA reactions seeded with the buffy coat from two of the vCJD cases (figure 8B and 9A) However, even after these six PMCA rounds, no PrP^res^ was detected in reactions seeded with buffy coat prepared from the third vCJD affected patient.

The rarity of blood samples collected in vCJD affected patients and the lack of samples from infected patients at preclinical stage of the disease are two major limitations for the development and performance assessment of vCJD blood detection assays. To model the capacity of this assay to detect lower amounts of blood vCJD agent (as expected in patients at preclinical stage) a ten-fold dilution series of WBC and BC samples from the three positive vCJD patients was made.

Using the WBC sample from the French vCJD affected patient, three amplification rounds allowed PrP^res^ detection in one out of two PMCA reactions seeded with material equivalent to 0.05 µL of starting whole blood (figure 9A). Similarly, after three PMCA rounds, PrP^res^ was detected in reactions seeded with 10^−3^ to 10^−5^ diluted buffy coat homogenates from two UK vCJD affected patients (figure 9B). Under the assumption that 3.5 mL of whole blood were used to prepare these BC (see method), these results indicate that less than 0,5 nL of whole blood equivalent material was sufficient to detect endogenous vCJD agent in the blood of these two patients.

Finally, the WBC homogenate from the French vCJD affected patient were mixed with WBC from either eleven (8 different pools), twenty-three (4 different pools), forty-seven (2 different pools) or ninety-five (1 pool) healthy donors plus the WBC from the vCJD affected patient (figure 9C). After three PMCA rounds, reactions seeded using a pool constituted with up to forty-seven healthy donors plus the vCJD affected patient's WBC were PrP^res^ positive. All the reactions seeded with pools containing only WBC from healthy donors were negative.

## Discussion

Cell free conversion assays have been extensively used to investigate PrP^Sc^ induced PrP^C^ to PrP^Sc^ conversion. Combinations of PrP^Sc^ and PrP^C^ from different species have provided insight into the molecular basis for barriers to the transmission of TSEs between species (species barriers) and same-species hosts with different PrP genotypes (polymorphism barriers). Results obtained in this system, indicate that the reactions between PrP^Sc^ and PrP^C^ molecules of the same sequence are more efficient than heterologous sequence conversion. These results provided strong support for the concept that the sequence specificity in the conversion of PrP^C^ to PrP^Sc^ modulates the interspecies or intraspecies transmissibility of TSE agents [40]–[46].

The results obtained here when amplifying by PMCA vCJD/BSE agents originating from different species are not fully consistent with those findings. The observation that human PrP^C^ substrate support better PrP^Sc^ amplification when seeded with human vCJD agent than with any other source of vCJD/BSE agents, and that the murine PrP^C^ substrate was poorly efficient at amplifying non-murine vCJD/BSE agents, concur with the general conclusions derived from cell free conversion assay. However, the fact that whatever the considered source of vCJD/BSE agent (human, bovine, porcine etc…), the Q_171_ ovine PrP^C^ substrates provide better amplification than homologous PrP^C^ sequence substrates was unexpected.

PrP^Sc^ amplification levels in cell-free conversion assays remain very limited. This is a likely consequence of the fact that the newly formed PrP^Sc^ has either no or very limited seeding activity in this type of assay. In PMCA each sonication cycle is believed to create new seeding sites, including in the bulk of newly converted PrP^Sc^. These new seeds have the same amino acid sequence as the PrP^C^ substrate and therefore the efficacy of the PrP^C^ conversion could be enhanced. These differences might explain the discrepancies between our results and those previously reported using cell free conversion assay.

In addition, it is worth noting that whereas conventional mice are poorly susceptible to sporadic Creutzfeldt Jakob, they propagate variant CJD isolates prepared from patients displaying identical (Methionine homozygous at codon 129) PrP^C^ sequence [47]. This illustrates that rather than depending solely on the donor/recipient host PrP sequence homology the capacity of a prion to propagate efficiently in a host and in PMCA is also directly dependant of its strain properties. Similarly while human PrP substrate supported amplification of BSE adapted in ARQ sheep in PMCA, it did not allowed the amplification of ARQ sheep scrapie [20], [48]. This phenomenon could also contribute to an explanation for the results we obtained when amplifying vCJD/BSE by PMCA in different substrates.

Whether, at the molecular level, the species specificity of PMCA faithfully mimics the species barrier as observed in ‘living hosts’ remains to be thoroughly assessed. Interestingly prion strains amplified by PMCA using a homologous PrP amino acid sequence as the substrate share identical biological properties to the parental strain, e.g. in bioassay [49]. In addition, propagation of a prion by PMCA using a substrate with a heterologous PrP sequence, can result in an evolution of its strain properties identical to that observed after *in vivo* propagation of this strain in the heterologous host (*i.e.* PMCA can reproduce the transmission barrier phenomenon) [50]. Here, the vCJD/BSE agent amplification obtained with different PMCA PrP^C^ substrates paralleled to some extent the propagation efficiency already reported *in vivo*. For instance, ovine BSE propagates with an apparently similar efficiency to cattle BSE prions in bovine transgenic mice (tgBov) [36] and with an higher efficiency in human transgenic mice (tg650) [51]. BSE/vCJD agents propagate with little or no transmission barrier in transgenic mice expressing the ovine ARQ PrP [33], [52], [53] and can be passaged in those expressing the ovine VRQ PrP variant (tg338 mice) [54].

However, in our opinion, there are still missing elements to establishing whether the PMCA amplification efficiency of an isolate/substrate combination is systematically correlated to the corresponding bioassay sensitivity. In this context, a end-point titration of the vCJD/BSE isolates used in the different transgenic PrP mouse lines (tga20, tgBov, tg338 and tgShXI) has been initiated.

Despite the limited number of vCJD clinical cases observed so far (n = 177) in the United Kingdom, the most recent epidemiological studies indicate that, in this country, 1 out 2000 people could carry the vCJD agent. In the absence of validated vCJD screening assay, UK like most of the developed countries apply systematic measures aiming at mitigating the blood borne transmission risk of the disease. These measures represent a substantial cost and increase the difficulty met by the blood banking system to provide certain blood products. In that context the added value from a vCJD blood detection assay is obvious.

The absence of human blood samples that would have been collected in infected individuals at asymptomatic stage of the disease represents a major limitation for developing and validating such assay. Using the two animal models that are considered as the most relevant for vCJD agent infection (sheep and primates), our study demonstrates that an assay based on the *in vitro* amplification of BSE/vCJD by PMCA allows an early and specific detection of infected animals.

The blind testing of two sample panels, that included a limited number of vCJD cases (n = 4) and a substantial number of healthy controls (n = 141), provides evidence that PMCA can be used for detecting vCJD agent in blood in human. These results also demonstrate the very high sensitivity of the PMCA method for detecting the endogenous vCJD agent associated to WBC/buffy coat in three vCJD affected patients, as detection can be possible with the equivalent of 0.5 nL of infected whole blood. However, despite its sensitivity, our assay failed to amplify PrP^res^ in the reactions seeded with buffy coat from one of the vCJD affected patient.

This failure might be the consequence of several non-exclusive phenomena. First, it might be due to the sample processing. Indeed our experiments in vCJD infected primates clearly highlighted that buffy coat can contain PMCA inhibitors

Alternatively, this negative result might be due to a lower or absent prionemia in certain vCJD affected patients. This explanation would fit with the results reported by the MRC unit in the UK using a different vCJD blood detection assay. Rather than amplifying abnormal PrP, this assay is based on the capture of non PK digested disease-associated PrP on a solid-state binding matrix. Like our PMCA method, the MRC vCJD blood assay displayed an excellent analytical sensitivity and specificity. However about a third of the vCJD blood samples tested so far were score negative (6 out the 21 vCJD affected cases) [26], [55], [56]. The idea of a lower/absence of prionemia in certain vCJD cases is also indirectly supported by the observations recently reported by Mead et al. This author reported that in a vCJD affected patient that was negative using the MRC vCJD blood detection assay, the lympho-reticular tissues displayed unusually low PrP^Sc^ accumulation levels [57]. Beyond this, a low level or an absence of infectivity in the blood of certain vCJD infected patients could also explain the lack of disease transmission observed so far in certain patients who received blood from donors who later developed vCJD [58].

To date, the presence of vCJD endogenous infectivity in human blood has been formally established (bioassay) in a single affected patient [12]. In that context, measuring through bioassay the infectivity level in blood from a panel of vCJD affected patients (including if possible vCJD blood samples that were scored negative for PrP^Sc^ presence) would be highly valuable.

For more than a decade PMCA has been reputed to be a highly sensitive but unreliable technique [59]. Even if there is still a need for standardisation of protocols and for an optimisation of hardware, in our opinion, the reliability of the technique has now reach an acceptable level. Moreover, the recent progress in the miniaturisation of the method [60] and the demonstration that brain homogenate can be replaced by cell lysate [61] should further facilitate the use of this technique.

Over the last few years alternative methods to PMCA for *in vitro* amplification and detection of prions have been developed. The quaking induced conversion (QuIC) and the real time QuIC (RT-QuIC) are based on fibrillation of a recombinant PrP (rec-PrP) substrate triggered by the presence of a minute amount of PrP^Sc^ [62], [63]. QuIC already allowed highly sensitive detection of abnormal PrP^Sc^ in various biological fluids and some studies reported its capacity to detect brain derived vCJD PrP^Sc^ in plasma [64]–[66]. The possibility of using bacterial rec-PrP and the apparent simplicity of these methods are quite attractive. However, at this stage, in case of a positive reaction, the assay does not offer the opportunity to confirm directly the nature of the TSE agent that triggered conversion. In contrast, since PrP^Sc^ amplified with PMCA has all the biochemical characteristics of the original seed (in our case BSE/vCJD) this method allows the direct identification of the vCJD agent signature in positive reactions.

Despite all the remaining difficulties, the results obtained so far by two different methodologies (PMCA as presented here and the abnormal PrP capture), and the rapid progress of QuIC derived technologies, allow potential new possibilities for vCJD screening and the prevention of its iatrogenic transmission.

## Supporting Information

Figure S1 **PrP^C^ in PMCA substrate prepared using brain from different mouse lines.** Total proteins from an aliquot of each type of PMCA substrate were quantified and five µg of proteins were mixed with an equal volume of 2X Laemmli's buffer before Western blotting and PrP^C^ probing using Sha31 antibody (epitope YEDRYYRE).
(TIF)
