# Supplementary figures and images for: Preclinical Detection of Variant CJD and BSE Prions in Blood
Source: PLoS Pathog. 2014 Jun 12;10(6):e1004202. doi: 10.1371/journal.ppat.1004202 (PMC4055790; doi:10.1371/journal.ppat.1004202)

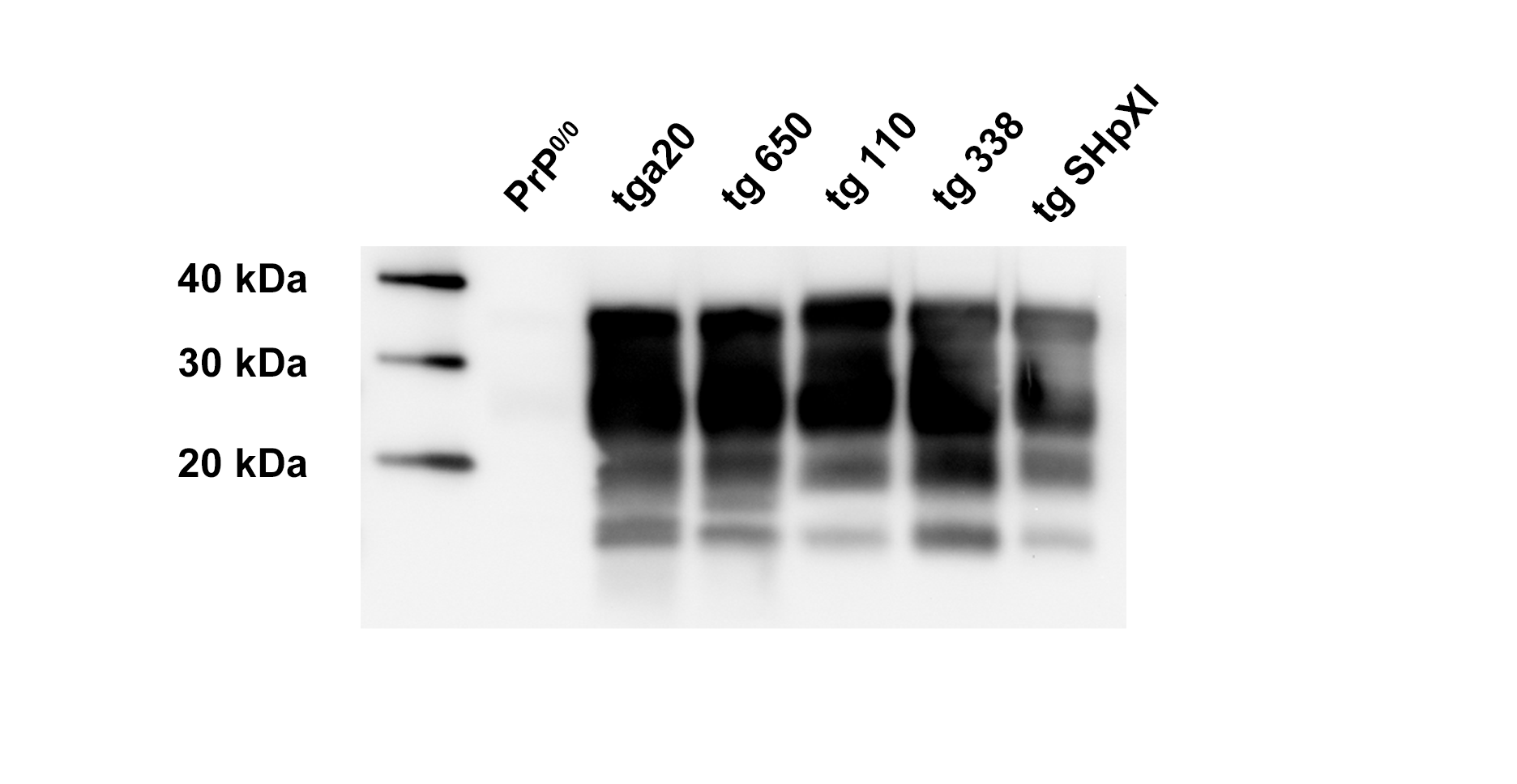

Supplement: Figure S1 — PrPC in PMCA substrate prepared using brain from different mouse lines. Total proteins from an aliquot of each type of PMCA substrate were quantified and five µg of proteins were mixed with an equal volume of 2X Laemmli's buffer before Western blotting and PrPC probing using Sha31 antibody (epitope YEDRYYRE). (TIF) [file ppat.1004202.s001.tif]
